# The complete plastome genome sequence of *Cynanchum otophyllum* (Asclepiadaceae), a unique medicinal species in China

**DOI:** 10.1080/23802359.2023.2290850

**Published:** 2024-03-11

**Authors:** Bosen Li, Haobin Wang, Junling Zhang, Bin Qiu, Congwei Yang, Yaqiong Li

**Affiliations:** College of Traditional Chinese, Medicine, Yunnan University of Traditional Chinese Medicine, China

**Keywords:** *Cynanchum otophyllum*, complete plastome genome, asclepiadaceae, aunique medicinal plant in China

## Abstract

*Cynanchum otophyllum* Schneid is an important medicinal plant in China. In this paper, the chloroplast genome of *C. otophyllum* was sequenced based on high-throughput technology, and the chloroplast genome structure characteristics and phylogenetic relationship of *C. otophyllum* were analyzed. The results showed the complete plastome genome size of *C. otophyllum*is 160,874bp, including one small single copy (SSC, 19,851bp) and one large single copy (LSC, 92,009bp) regions isolated by a pair of inverted repeat regions (IRs, 24,507bp). The whole plastome genome including 84 protein encoding genes, 8 rRNA and 37 tRNA. Based on the phylogenetic topologies, *C. otophyllum* shows close association with additional *Gomphocarpus* and *Asclepias* genus. This study contributes to an enhanced understanding of the genetic information of *C. otophyllum* and provides a theoretical basis for the development of molecular markers and phylogeographic of the species, as well as for constructing the phylogenetic tree of Asclepiadaceae.

## Introduction

Chloroplasts are photosynthetic plastids in green plants. The plastome genome (cpDNA) is generally a circular double-stranded and typical tetrad junction. The chloroplast genome contains a large single copy region (LSC), a small single-copy region (SSC), and two reverse repeat regions with the same sequence and opposite directions (Jansen et al. 2005). In angiosperms, the plastome genome is mostly maternally inherited. Compared with the nuclear genome and ribosomal genome, the plastome genome is smaller, only 120–220 kb in higher plants.Because the plastome genome contains a small number of functional genes and low mutation rate, it is often used as an excellent genetic variation marker in plant phylogenetic and phylogenetic geography (Palmer 1985; Wolfe et al. 1987；Wang and Hong 1997). Based on the analysis of the structure and composition of the plastome genome of species, it will lay a foundation for further research on bioinformatics and population genetics (Guisinger et al. 2011, Yu et al. 2022; Liang et al. 2019).

**Figure 1.**
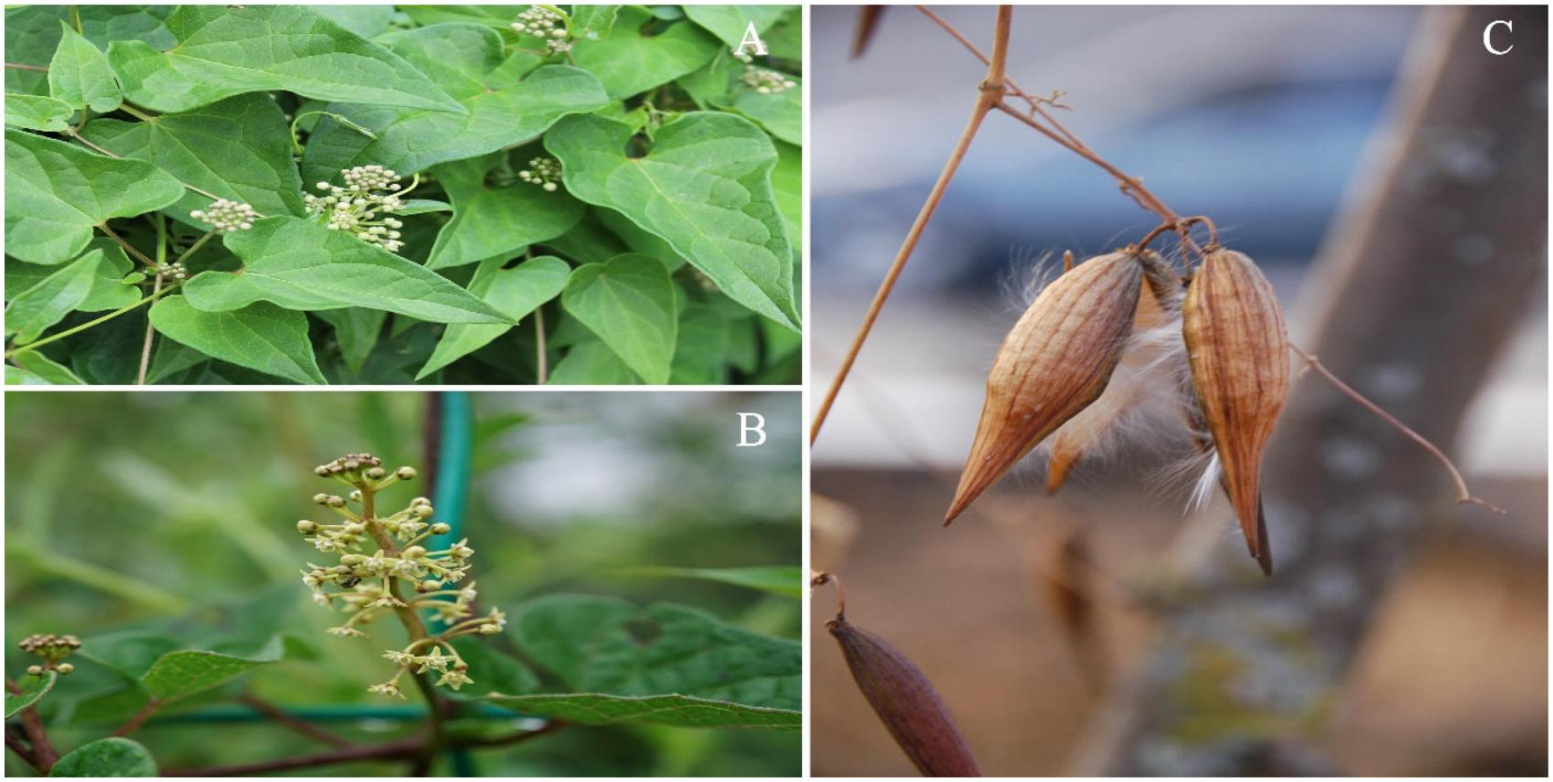
The morphological characteristics of *Cynanchum otophyllum* A, B, C showing the photo of plants, flowers and fruits respectively (photos taken by Congwei Yang).

*Cynanchum otophyllum* Schneid. (in Sarg. Pl. Wils. 1916), a perennial herbaceous vine of the *Cynanchum* genus of the Asclepiadaceae family (Figure 1), is a Chinese unique species, distributed within an extremely limited range only in China, at altitude range of 1500–2800 m (Li et al. 1995). It has been found to contain several components with bio-active effects, including steroids saponin, benzene derivatives, organic acid, monosaccharide,oligosaccharide, and trace mineral elements (Shen et al. 2014; Yang et al. 2015; Li et al. 2016). Its rhizomes areregarded as the medicinal parts of the plant, and have been used extensively to treat diseases such as epilepsy, hysteromyoma, lumbar bone pain, tubercle and nourishing strong (Kuang et al. 1991; Zhan et al. 2019). This species is one of the most important Chinese traditional herb also called Qingyangshen, it had been widely used in Chinese nationalities of Bai, Naxi and Yi to treat epilepsy and anti-hepatitis for a long time. *C. otophyllum* has been recognized as the endangerd medicinal plant because resources of this herb are diminishing due to uncontrolled harvesting (Wang et al. 2009). Therefore, it is necessary for us to learn more about its genetic data and pay more attentionto it. Notably, the chloroplast genome-wide for *C. otophyllum* would help to conserve the precious natural populations.

## Materials and methods

In this study, fresh leaves of *C. otophyllum* were collected from Shi lin County, Kunming City, Yunnan Province (24°77.176′ N; 103°35.201′E), and a voucher specimen of *C. otophyllum* was deposited in the Herbarium of Yunnan College of Traditional Chinese Medicine (Certificate No.: CO20220812: Li Yaqiong; liyaqiong6 @ 126.com). Total genome DNA was extracted with CTAB method from the fresh and healthy leaves of *C. otophyllum* (Li et al. 2013). Illumina paired-end libraries (150 bp read length) were prepared from sheared genomic DNA (fragment size ≤800 bp) and the complete plastome genome sequencing was taken on an Ilumina Hiseq × Ten platform (Illumina, San Diego, CA, USA). The raw reads with the adapters trimmed were filtered by quality with Phred scores of 30 or less implemented in the CLC-quality trim tool(Li et al. 2022), and the filtered reads were assembled using GetOrganelle v1.7.5 (Jin et al. 2020). Reference-guided connecting and annotation was subsequently conducted using Geneious 9.1.4 (Kearse et al. 2012) and Bandage 0.8.1 (Wick et al. 2015). In addition, the GeSeq (https://chlorobox.mpimp-golm.mpg.de/geseq.html) (Tillich et al. 2017) was utilized for annotation with the reference of *Cynanchum wilfordii* (KT220733) as a reference sequence, and the boundaries were manually corrected. Then a Maximum likelihood (ML) was performed to estimate the phylogeny for 14 plastome sequences of Asclepiadaceae species, with two *Gomphocarpus* R. Br. and *Asclepias* Linn. species as outgroups. ML analysis was implemented using RAxML v8.1.11 (Stamatakis 2014), so as to examine the *C. otophyllum* position in the phylogenetic tree.

## Result

**Figure 2.**
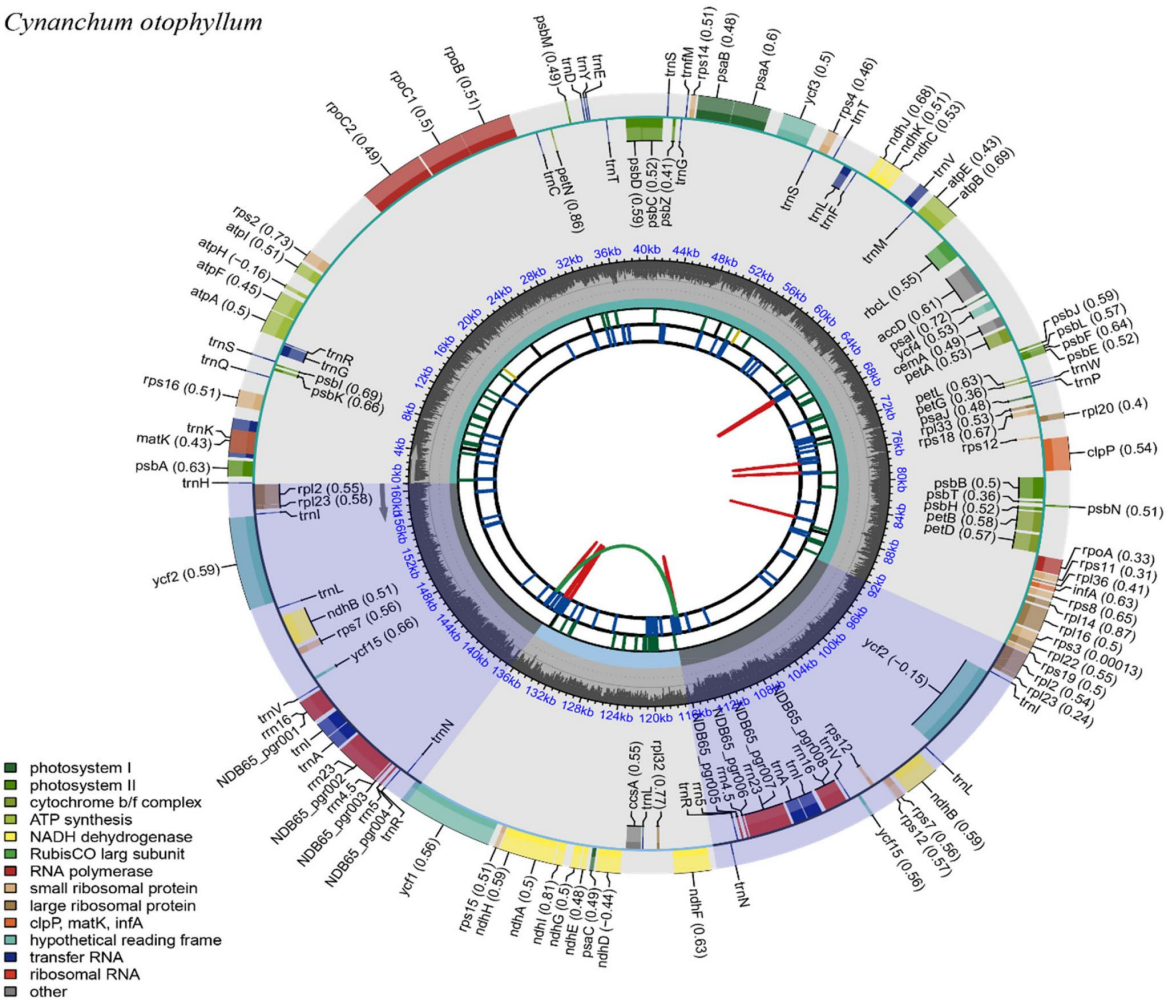
Chloroplast complete genome map of *Cynanchum otophyllum*. The map consists of several circles, each with the following information from the center outward: the circles closest to the center are indicated by red and green arcs for forward and reverse repeats, respectively. The second and third circles are indicated by short bars for tandem repeats and microsatellite sequences, respectively. The fourth circle indicates the positions of the LSC, SSC, IRA, and IRB regions, respectively. The fifth circle indicates the GC content. The outer circle indicates the function of the gene. Different colors are used to show different functional categories, as shown in the lower left of the picture.

**Figure 3.**
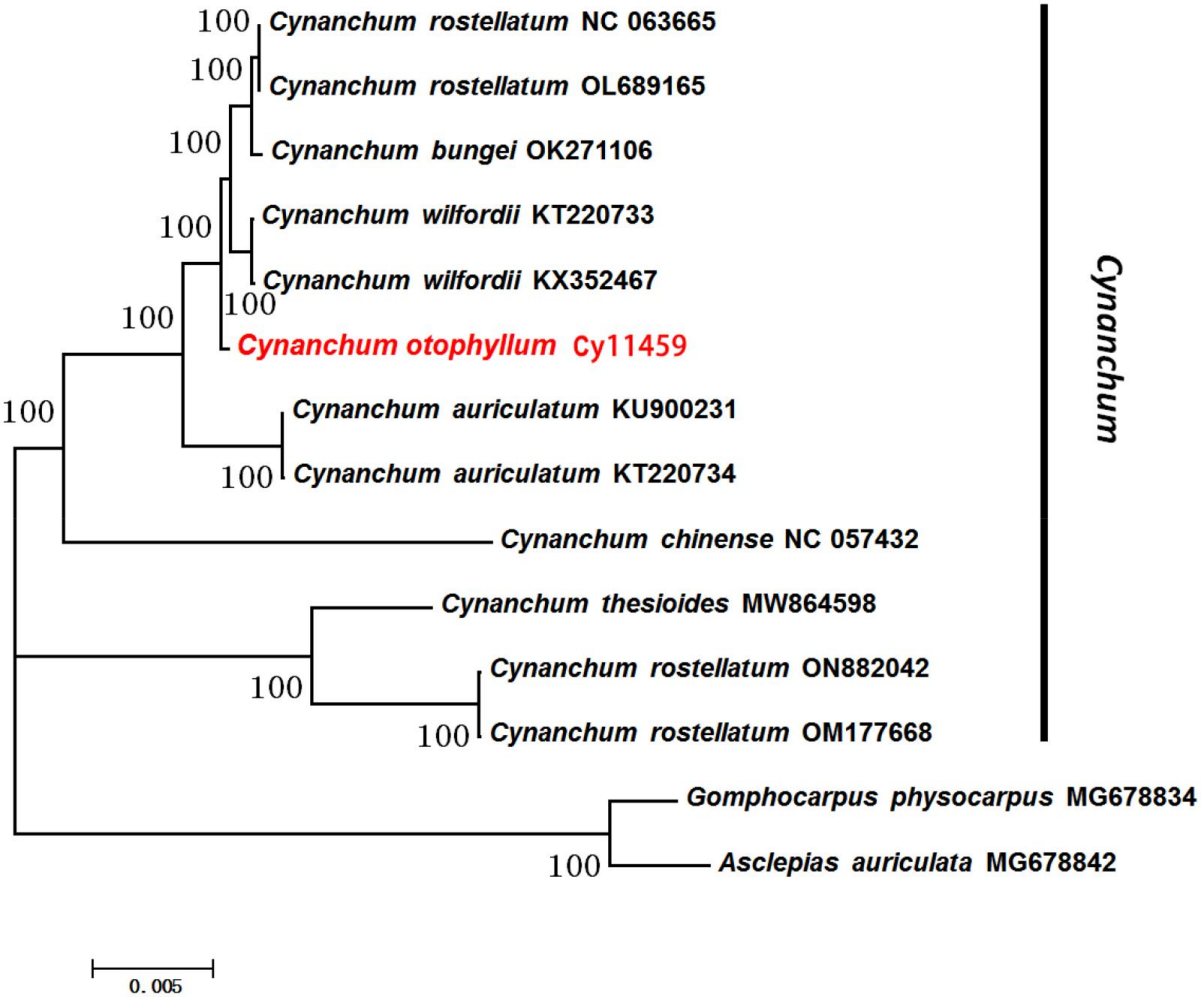
Fourteen plastome genomes were used for constructing maximum likelihoob (ML, bootstrap repeat is 1000). the sequences used for the tree structure are coding sequences, and the bootstrap support values are shown on the nodes. The used sequences and their references were listed in Table S1.

The complete plastome genome of *C. otophyllum* is a typical circular double-stranded DNA molecule with a length of 160,874 bp. The cp genome has the usual quadripartite structure of most angiosperms (Figure 2), containing a small single copy (SSC) region of 19,851 bp and a large single copy (LSC) region of 92,009 bp, which is separated by a pair of inverted repeat (IRs) regions of 24,507 bp and the base compositions of the cp genome were uneven (30.7% A,19.2% C, 18.6% G, and 31.5% T). The overall GC and AT content of the whole genome is 37.80% and 62.20%, respectively. GC content in the IR region (43.3%) is higher than that in the SSC region (32.0%) and LSC region (36.1%). A total of 129 genes are annotated, including 84 protein-coding genes (PCGs), eight ribosomal RNA genes (rRNAs), and 37 transfer RNA genes (tRNAs). In total, seventeen genes replicate in the IR region, repeating inversely with each other, including seven tRNA genes (trnA-UGC, trnI-CAU, trnI-GAU, trnL-CAA, trnN-GUU, trnR-ACG, trnV-GAC), six PCGs (rpl23, rpl2, rps7, ycf15, ycf2, ndhB), and four rRNA genes. Fourteen plastome genomes were used for constructing maximum likelihoob (bootstrap repeat is 1000). According to the phylogenetic trees, a monophyletic clade is formed among *C. otophyllum* together with the remaining *Cynanchum* species in the *Cynanchum* genus, and the bootstrap support value is 100% (Figure 3).

Specifically, the complete plastome genome for *C. otophyllum* would help to understanding the genetic information. The chloroplast genome structure of *C. otophyllum* was reported for the first time, which enriches the gene diversity, and this study provided new information for the phylogenetic relationship of the *Cynanchum* family.

## Supplementary Material

Supplemental Material

Supplemental Material

Supplemental Material

Supplemental Material

Supplemental Material

## Data Availability

The *Cynanchum otophyllum* complete plastome sequence has been stored in GenBank with accession number OQ587923. All the information can be found on the website (https://www.ncbi.nlm.nih.gov/). The associated BioProject, SRA, and Bio-Sample numbers are PRJNA947816, SRR23945207, and SAMN33867229, respectively. The data were collected without violationof the protection of human subjects, or other valid ethical, privacy, or security concerns.
