# Supplementary figures and images for: The complete plastome genome sequence of Cynanchum otophyllum (Asclepiadaceae), a unique medicinal species in China
Source: Mitochondrial DNA B Resour. 2024 Mar 11;9(3):318–21. doi: 10.1080/23802359.2023.2290850 (PMC10930142; doi:10.1080/23802359.2023.2290850)

# Trans-splicing Genes

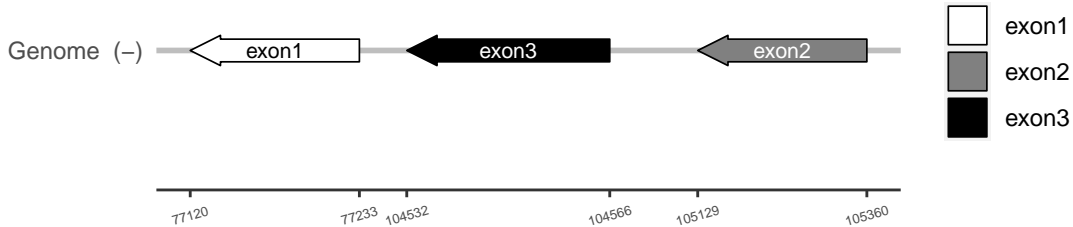

Supplement: Supplemental Material [file TMDN_A_2290850_SM6720.pdf]

# Cis-splicing Genes

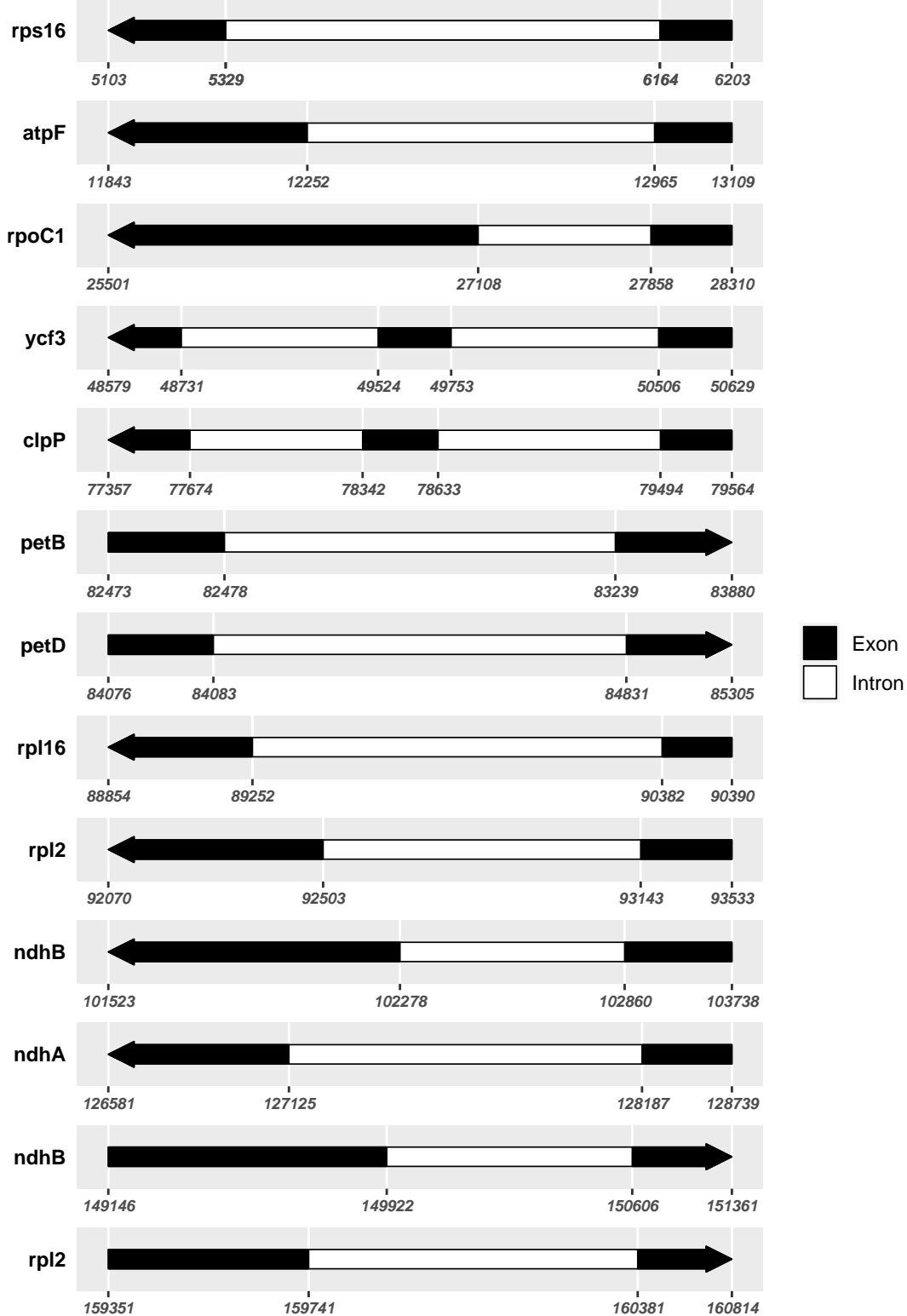

Supplement: Supplemental Material [file TMDN_A_2290850_SM6719.pdf]

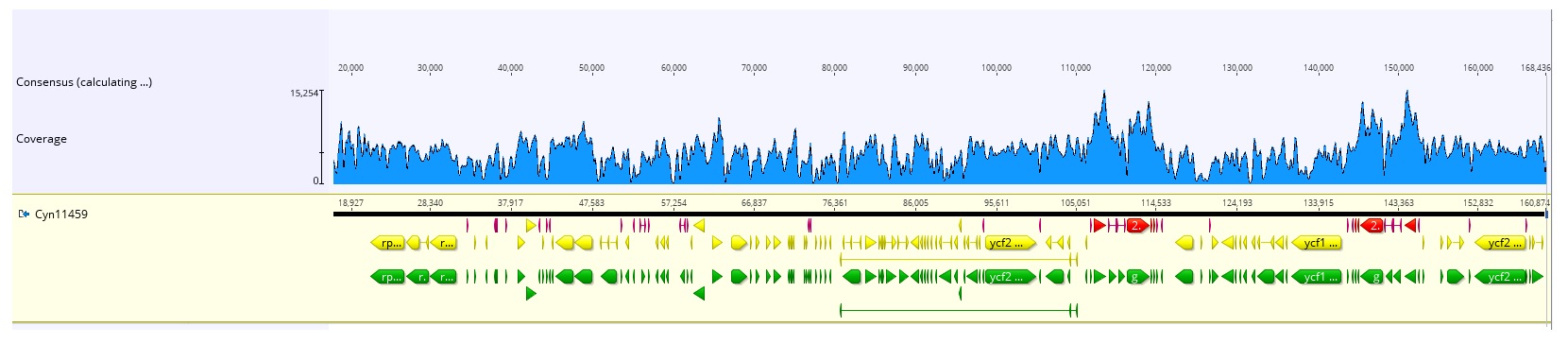

Supplement: Supplemental Material [file TMDN_A_2290850_SM6717.jpg]

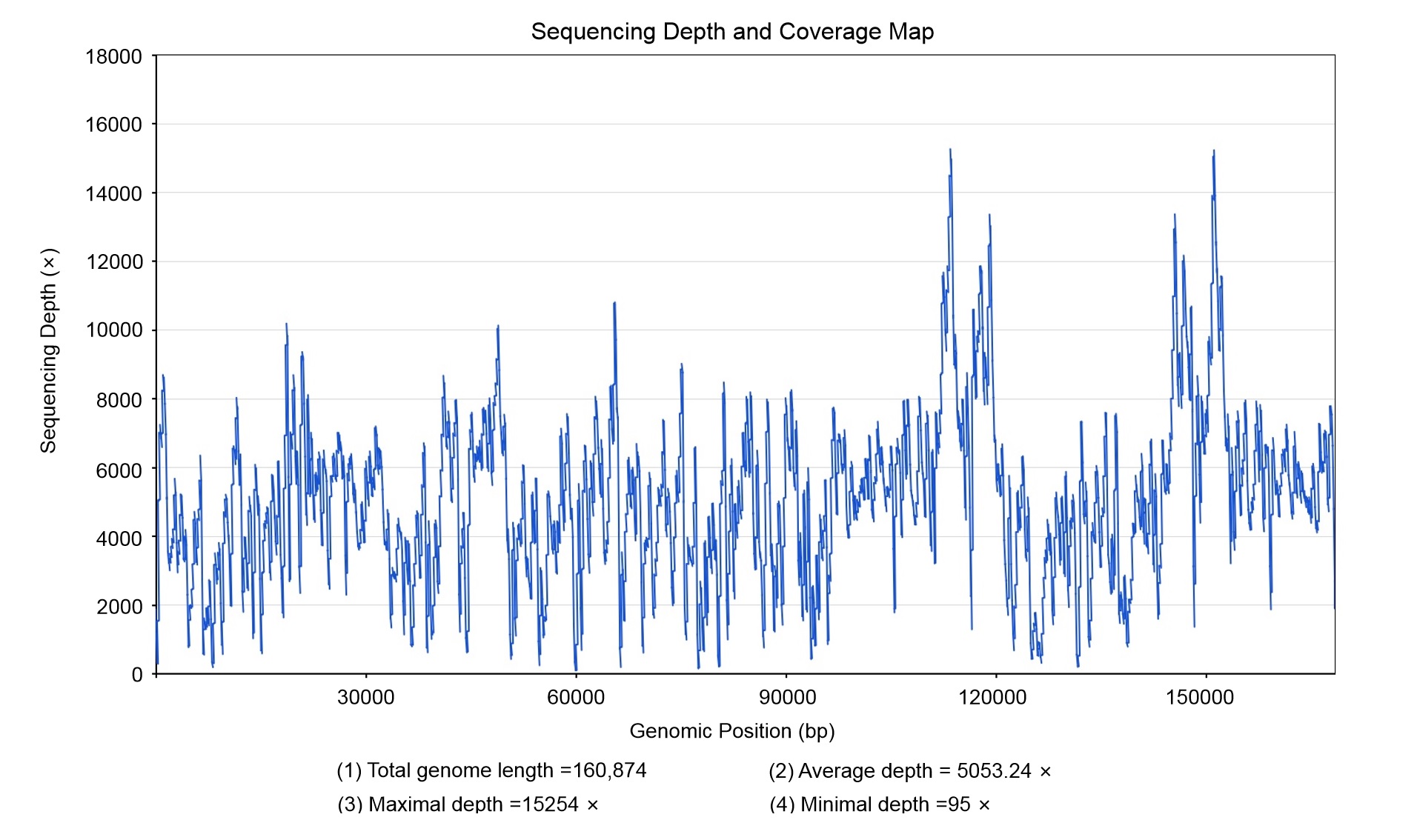

Supplement: Supplemental Material [file TMDN_A_2290850_SM6716.jpg]
