# Supplementary material for: The complete plastome genome sequence of Cynanchum otophyllum (Asclepiadaceae), a unique medicinal species in China
Source: Mitochondrial DNA B Resour. 2024 Mar 11;9(3):318–21. doi: 10.1080/23802359.2023.2290850 (PMC10930142; doi:10.1080/23802359.2023.2290850)
Supplement: Supplemental Material [file TMDN_A_2290850_SM6718.docx]

Table S1. Accession numbers and publications of Cynanchum otophyllum and other 13 species

| Species | Accession Number | Reference |
| --- | --- | --- |
| *Cynanchum rostellatum* | NC063665.1 | Not available |
| *Cynanchum rostellatum* | OL689165.1 | Lixin et al. 2022 |
| Cynanchum bungei | OK271106.1 | Pei et al. 2022 |
| *Cynanchum wilfordii* | *KT220733.1* | Park et al. |
| *Cynanchum wilfordii* | *KX352467.1* | Park et al. |
| ***Cynanchum otophyllum*** | *Cy11459* | *This study* |
| *Cynanchum rostellatum* | OM177668.1 | Lee SH et al. 2022 |
| *Cynanchum auriculatum* | KU900231.1 | Chen and Zhang 2022 |
| *Cynanchum auriculatum* | KT220734.1 | Jang et al.2016 |
| *Cynanchum chinense* | NC057432.1 | Not available |
| *Cynanchum thesioides* | MW864598.1 | Peng Kang et al. 2021 |
| *Cynanchum rostellatum* | ON882042.1 | Fishbein et al.2018 |
| *Cynanchum rostellatum* | OM177668.1 | [Sae Hyun Lee](https://www.tandfonline.com/author/Lee%2C+Sae+Hyun) et al. 2022 |
| *Gomphocarpus physocarpus* | MG678834.1 | Fishbein et al.2018 |
| *Asclepias auriculata* | MG678842.1 | Not available |

Lixin Pei, Shengnan Shu, Baoyu Ji & Ning Cui 2022 Complete sequence of Cynanchum rostellatum (Apocynaceae: Asclepiadoideae) chloroplast genome and its phylogenetic analysis, Mitochondrial DNA Part B, 7:7, 1395-1397.

Pei L, Shu S, Ji B, Cui N. 2022. Complete sequence of Cynanchum rostellatum (Apocynaceae: Asclepiadoideae) chloroplast genome and its phylogenetic analysis. Mitochondrial DNA Part B. 7(7):1395–1397

Park H-S, Kim K-Y, Kim K, Lee S-C, Lee J, Seong RS, Shim YH, Sung SH, Yang T-J. 2016. The complete chloroplast genome sequence of an important medicinal plant Cynanchum wilfordii (Maxim.) Hemsl. (Apocynaceae). Mitochondrial DNA Part A. 27(5):3747–3748.

Lee SH, Jang W, Kim E, et al. The complete plastome of Cynanchum rostellatum (Apocynaceae), an indigenous plant in Korea. Mitochondrial DNA B Resour. 2022;7(12):2035-2039. Published 2022 Dec 9.

Chen G, Zhang X. 2022. The complete chloroplast genome of Chinese medicinal herb Cynanchum chinense R. Br. (Apocynaceae) and its phylogenetic position. Mitochondrial DNA Part B. 7(4):598–599.

Woojong Jang, Kyu-Yeob Kim, Kyunghee Kim, Sang-Choon Lee, Hyun-Seung Park, Junki Lee, Rack Seon Seong, Young Hun Shim, Sang Hyun Sung & Tae-Jin Yang (2016) The complete chloroplast genome sequence of Cynanchum auriculatum Royle ex Wight (Apocynaceae), Mitochondrial DNA Part A, 27:6, 4549-4550

Peng Kang, Yuqian Guo, Yiyue Zhang & Yuqing Wei (2021) The complete chloroplast genome sequence of medicinal plant: Cynanchum thesioides (Asclepiadaceae), Mitochondrial DNA Part B, 6:9, 2592-2593

Sae Hyun Lee, Woojong Jang, Eunbi Kim, Jiseok Kim, Haiguang Gong, Jong-Soo Kang, Hyeonah Shim, Jee Young Park & Tae-Jin Yang (2022) The complete plastome of Cynanchum rostellatum (Apocynaceae), an indigenous plant in Korea, Mitochondrial DNA Part B, 7:12, 2035-2039

Fishbein M, Livshultz T, Straub SCK, Simões AO, Boutte J, McDonnell A, Foote A. Evolution on the backbone: Apocynaceae phylogenomics and new perspectives on growth forms, flowers, and fruits. Am J Bot. 2018 Mar;105(3):495-513
